# Association between a *SLC23A2* gene variation, plasma vitamin C levels, and risk of glaucoma in a Mediterranean population

**Published:** 2011-11-17

**Authors:** Vicente Zanon-Moreno, Lucia Ciancotti-Olivares, Jeronimo Asencio, Pedro Sanz, Carolina Ortega-Azorin, Maria D. Pinazo-Duran, Dolores Corella

**Affiliations:** 1 Genetic & Molecular Epidemiology Unit, Department of Preventive Medicine & Public Health, School of Medicine, University of Valencia, Valencia, Spain; 2 CIBER Fisiopatología de la Obesidad y Nutrición, University of Valencia, Valencia, Spain; 3 Department of Preventive Medicine & Public Health, Dr. Peset University Hospital, Valencia, Spain; 4 Ophthalmology Research Unit “Santiago Grisolia,” Dr. Peset University Hospital, Valencia, Spain

## Abstract

**Purpose:**

Several dietary factors have been associated with glaucoma. Among them, dietary antioxidant intake (i.e., vitamin C and vitamin A) in association with glaucoma has been analyzed, but with mixed results. Genetic factors may play a role in modulating the effect of dietary antioxidant intake on glaucoma; however, nutrigenetic studies in this field are scarce. Our aim was to study the association between selected polymorphisms in key proteins related to vitamin C and vitamin A concentrations and primary open-angle glaucoma (POAG).

**Methods:**

We performed a case-control study matched for age, sex, and bodyweight. We recruited 300 subjects (150 POAG cases and 150 controls) from a Mediterranean population and determined the plasma concentrations of vitamin C and vitamin A for each subject. We selected the following single-nucleotide polymorphisms (SNPs) in genes related to vitamin A and vitamin C concentrations: rs176990 and rs190910 in the retinol-binding protein 1 (*RBP1*) gene; and rs10063949 and rs1279683 in the Na^+^-dependent L-ascorbic acid transporters 1 and 2, respectively (encoded by the *SLC23A1* and *SLC23A2* genes).

**Results:**

We found a statistically significant association between the rs1279386 (A>G) SNP in *SLC23A2* and POAG risk. In the crude analysis, homozygous subjects for the G allele (GG subjects) had higher risk of POAG than other genotypes (OR: 1.67; 95% CI: 1.03–2.71). This association remained statistically significant (p=0.010) after multivariate adjustment for potential confounders. We also found that POAG patients had lower plasma vitamin C concentrations than control subjects (9.9±1.7 µg/ml versus 11.7±1.8 µg/ml, p<0.001). Moreover, we consistently detected a significant association between the rs1279386 SNP in *SLC23A2* and plasma vitamin C concentrations: GG subjects had significantly lower plasma vitamin C concentrations than the other genotypes (9.0±1.4 µg/ml versus 10.5±1.6 µg/ml, p<0.001 in POAG cases and 10.9±1.6 µg/ml versus 12.1±1.8 µg/ml, p<0.001 in controls). The rs10063949 SNP in *SLC23A1* was not associated with either plasma vitamin C concentrations or POAG risk. Similarly, SNPs in *RBP1* were not associated with vitamin A concentrations or POAG risk.

**Conclusions:**

The rs1279683 SNP in *SLC23A2* was significantly associated with lower plasma concentrations of vitamin C and with higher risk of POAG in GG subjects.

## Introduction

Glaucoma is a group of diseases in which the optic nerve is damaged, leading to blindness. The most common form is primary open-angle glaucoma (POAG) [1]. The main cause of developing POAG is alteration in the eye tissues, of the anterior chamber, involved in intraocular pressure (IOP) regulation, resulting in aqueous humor (AH) outflow impairment and ocular hypertension (OHT) [2]. Many factors have been associated with an increased risk of developing this disease, including age, race, myopia, family history, and several single-nucleotide polymorphisms (SNPs) in the genes [3-8].

Among the non-genetic factors that may be associated with this optic neuropathy, those related to nutrition have recently been emerging, but their importance is still unknown. Recent studies suggest that nutritional factors may play a role in the pathogenic mechanisms of glaucoma [9,10], and that these factors could be related to modifications in the trabecular meshwork (TM; vitamin C, glutathione) and to optic atrophy (vitamin E) [11]. Because vitamins such as A and C might be involved in the progression of glaucomatous optic neuropathy, the nutritional status of patients is very important in maintaining good eye health [12,13]. Moreover, from a nutrigenetic point of view, SNPs can modulate the effect of dietary intake on POAG risk, but this field remains unexplored.

Vitamin A (retinol) is an essential nutrient in maintaining ocular health. This vitamin plays a key role in the development and proper functioning of vision (retinol helps maintain cells and eye tissues, such as the retina) and prevents eye diseases [14,15]. A lack of vitamin A can lead to serious ocular problems: loss of visual acuity after dark, dryness of the conjunctiva, inflammation of the eyelids, and corneal ulceration [16,17]. One of the genetic factors related to the metabolism and functions of vitamin A, cellular retinol-binding protein type 1 (*CRBP1*), which is encoded by the retinol-binding protein gene (*RBP1*), serves to safeguard retinol homeostasis [18].

Vitamin C is an important water-soluble antioxidant [19]. Its adequate consumption may prevent the development of eye diseases, especially degenerative ones, like glaucoma. With regard to this, a recent study found that serum concentrations of vitamin C were significantly lower in glaucoma patients than in healthy controls [20]. Plasma concentrations of vitamin C are determined by dietary intake, as well as by genetic factors. L-Ascorbic acid obtained from the diet is transported across the cell membrane by sodium L-ascorbic acid cotransporters (SVCTs). Two isoforms, SVCT1 (encoded by the *SLC23A1* gene) and SVCT2 (encoded by the *SLC23A2* gene), play central roles in the absorption and accumulation of vitamin C in many tissues [21,22].

Thus, the aim of this study was to investigate the association of selected SNPs in *RBP1*, *SLC23A1*, and *SLC23A2* with POAG and to test whether this potential effect was mediated by the association of these polymorphisms with plasma concentrations of vitamins A and C.

## Methods

### Subjects and study design

We performed a matched case-control study, in which the same number of POAG subjects and control subjects from a Mediterranean population were paired by gender, age (±2 years), and body mass index (BMI; categorized into normal weight, overweight, and obese subjects). We recruited 300 Caucasian participants (150 patients with POAG, 61 men and 89 women, with mean age 68±9 years; and 150 controls) from two health centers in the city of Valencia (Spain): the University Hospital Dr. Peset, Valencia (Department of Ophthalmology and Department of Preventive Medicine & Public Health) and the School of Medicine of the University of Valencia (Department of Preventive Medicine & Public Health). Review boards and ethical committees of the University of Valencia approved the protocols for this study, which complied with the Helsinki guidelines on human research. Informed consent forms were signed by all study participants.

Cases were subjects with POAG diagnosed by an ophthalmologist and were aged from 50 to 80 years. Controls were subjects without ocular diseases (POAG, cataracts, age-related macular degeneration, or severe myopia) and were matched with POAG cases by sex, age, and BMI. Cases with ocular diseases other than POAG, such as rickets, osteoporosis and osteomalacia, and/or aged outside the range of inclusion were excluded. Controls with rickets, osteoporosis or osteomalacia; aged outside the range of inclusion; and/or with a family history of glaucoma were excluded. POAG patients underwent a systematized ophthalmic examination, including slit lamp biomicroscopy, IOP measurement (with Goldmann applanation tonometry), best corrected visual acuity, visual field performance (using the 24–2 Swedish Interactive Threshold Algorithm [SITA; Humphrey Field Analyzer II; Carl Zeiss Meditec, Inc., Dublin, CA]), ocular imaging analysis (using optical coherence tomography [Stratus OCT; Carl Zeiss Meditec, Inc.]), and dilated stereoscopic fundus examination under a 78- diopters (D) lens with simultaneous optic disc photographs. Data on duration of the disease, family background, therapy, and various aspects of the ophthalmological examination were registered using a self-designed database. IOP was also measured in healthy controls. POAG cases and controls completed a questionnaire regarding socio-demographic, clinical, and lifestyle variables.

### Biochemical and genetic analysis

Global standards for patient biosample workflow, transport, storage, and proceedings were strictly followed. In both POAG cases and controls, whole blood samples were obtained from the antecubital veins of the participants under fasting conditions (10–11 h). Blood was collected into EDTA tubes (two 5-ml tubes per subject, one for DNA extraction and the other for obtaining plasma).

Genomic DNA was extracted from blood with the MagNA Pure LC DNA Isolation Kit (Life Sciences Corp., Branford, CT). Genotyping of the following SNPs was performed on a 7900HT Sequence Detection System (Applied Biosystems, Foster City, CA) using standard TaqMan fluorescent allelic discrimination assays: rs176990 and rs190910 in *RBP1*, rs10063949 in *SLC23A1*, and rs1279683 in *SLC23A2*.

Plasma concentrations of vitamin C were measured using the method of Li et al. [23]. Briefly, analyses were performed using Shimadzu Scientific Instruments (SSI, Columbia, MD) equipment with an LC-20AB delivery pump and a Coulochem III (ESA, Chelmsford, MA) electrochemical detector, under reversed phase conditions with a 4.6×250 mm, 5 µM YMC-Pack ODS-AQ column (Waters Corp., Milford, MA). The software used was LabSolutions 1.2 (SSI, Columbia, MD). Compounds were eluted over an 18-min runtime at a flow rate of 0.6 ml/min. The mobile phase consisted of methanol/150 mM chloroacetate (3:97, v/v) and 2mM disodium EDTA (pH adjusted to 3.0 with 10 N NaOH). Sample injection was 5 µl.

Vitamin A plasma concentrations were measured according to the method of Ortega et al. [24], with modifications. Briefly, analyses were performed in a Gilson Medical Electronics HPLC system (Middleton, WI) with an LC307 delivery pump, an LC142 electrochemical detector (325 nm), and a Shim-pack CLC-ODS C18 column (250 mm×6 mm). Compounds were eluted over a 20-min runtime at a flow rate of 1 ml/min. The mobile phase consisted of methanol/water (95:5, v/v), and sample injection was 50 µl.

### Statistical analysis

Pearson’s χ^2^ test was used to compare categorical variables and to check Hardy–Weinberg equilibrium (HWE) for the analyzed SNPs. The normal distribution of continuous variables was checked first. These variables were analyzed using the Unpaired Student’s *t*-test (2 groups), and the ANOVA test with post-hoc Bonferroni correction was used (for more than 2 groups) to compare mean differences among the groups. To estimate the risk of glaucoma associated with the genotypes, logistic regression models were fitted, and the odds ratios (OR) and their 95% confidence intervals (CI) were estimated. Univariate and multivariate regression models were also fitted, including control for potential confounders (sex, age, BMI, tobacco smoking, and alcohol drinking). Multivariate lineal regression models were used to adjust continuous variables (vitamin concentrations) for the potential confounders. The original significance level was set at a p-value of 0.05 by a two-tailed test, with the Bonferroni correction applied to compensate for multiple comparisons. This approach consists of using an adjusted alpha level equal to the original alpha level (0.05), divided by the number of SNPs analyzed. In our case, the adjusted alpha is 0.05/4=0.0125. The significance of each uncorrected test was assessed at this level. PASW software (version 17.0, SPSS, Inc., Chicago, IL) and the program Haploview 3.32 were used for statistical analysis.

## Results

The general characteristics of all 150 POAG patients and their paired controls are shown in Table 1. Call rates for the genotyped SNPs (rs176990 and rs190910 in *RBP1*, rs10063949 in *SLC23A1*, and rs1279683 in *SLC23A2*) were all 100%. Genotype distributions in the controls exhibited Hardy–Weinberg equilibrium (p=0.920, p=0.306, p=0.178, and p=0.195 for rs176990, rs190910, rs10063949, and rs1279683, respectively). In the POAG cases, all SNPs had p-values >0.05 except for rs10063949 (p=0.018). Thus, none of the four SNPs deviated from HWE in POAG cases after Bonferroni correction for multiple comparisons (p>0.05/4=0.0125).

**Table 1:** General characteristics of POAG cases and controls.

**Characteristics**	**Cases (n=150)**	**Controls (n=150)**	**p**
Females (%)	59.3	59.3	1.000
Age (years)	68±9	68±8	0.973
BMI (kg/m^2^)	27.5±4.7	27.4±4.9	0.801
Cup disk ratio	0.7	0.3	<0.001*
Smokers (%)	28	22	0.230
Alcohol consumers (%)	74	68	0.252

*Statistically significant (p<0.0125) differences between cases and controls. BMI: Body mass index.

Table 2 shows the genotype frequencies of the four SNPs in POAG cases and controls. For each SNP, we have first indicated the genotypes for the ancestral allele, which was the major allele only for rs10063949. In this population, the ancestral allele was the minor allele for the other three SNPs. After evaluation of the genotype distributions, and taking into account the p-value obtained in the comparisons between cases and controls, we grouped homozygous and heterozygous subjects for the ancestral allele and compared this group with homozygous subjects for the non-ancestral (variant) allele. OR and 95% CI were estimated for each SNP. The group of homozygous and heterozygous subjects for the ancestral allele was considered as the reference category. Crude and multivariate adjusted p-values were estimated (Table 2). The crude risk (OR) of POAG associated with the GG genotype of *SLC23A2* in comparison with AA + AG subjects was 1.67 [95% CI (1.03–2.71), p=0.038]. After adjustment for potential confounders (sex, gender, BMI, tobacco smoking, and alcohol consumption), this estimation reached statistical significance (p=0.010), taking into account the significance of the threshold p-value (p=0.0125) after the correction for multiple comparisons. We did not find any significant association for the other SNPs.

**Table 2:** Genotypic frequencies of the *RBP1*, *SLC23A1*, and *SLC23A2* polymorphisms in POAG cases and controls.

** **	** **	** **	**Genotype frequencies (%)**						** **	** **	** **	** **
** **	** **	** **	**Cases (n=150)**			**Controls (n=150)**			** **	** **	** **	** **
**Gene**	**SNP**	Alleles* (1/2)	**1/1**	**1/2**	**2/2**	**1/1**	**1/2**	**2/2**	**p1**	OR** (95%CI)	**p2**	**p3**
*RBP1*	rs176990	T/G	25%	43%	32%	19%	48%	33%	0.423	0.97 (0.60–1.57)	0.972	0.826
*RBP1*	rs190910	A/T	20%	52%	28%	15%	53%	32%	0.514	0.83 (0.50–1.36)	0.451	0.315
*SLC23A1*	rs10063949	T/C	34%	40%	26%	33%	44%	23%	0.727	1.19 (0.71–2.03)	0.501	0.552
*SLC23A2*	rs1279683	A/G	20%	41%	39%	17%	55%	28%	0.044	1.67 (1.03–2.71)	0.038	0.010***

*Allele 1 is the ancestral allele. Allele 2 is the variant allele. **Odds ratio (OR) and 95% confidence interval (CI) for homozygous subjects for the variant allele (2) compared with the reference category (homozygotes and heterozygotes for the ancestral allele). p1: Uncorrected p value obtained in the corresponding χ^2^ test for the comparison of the genotype distribution between cases and controls. p2: Uncorrected p value obtained in the crude logistic regression analysis. p3: Uncorrected p value obtained in the multivariate logistic regression after adjustment for sex, age, BMI, tobacco smoking, and drinking. ***Statistically significant p value (p<0.0125) after the Bonferroni correction for multiple comparisons.

**Figure 1:**
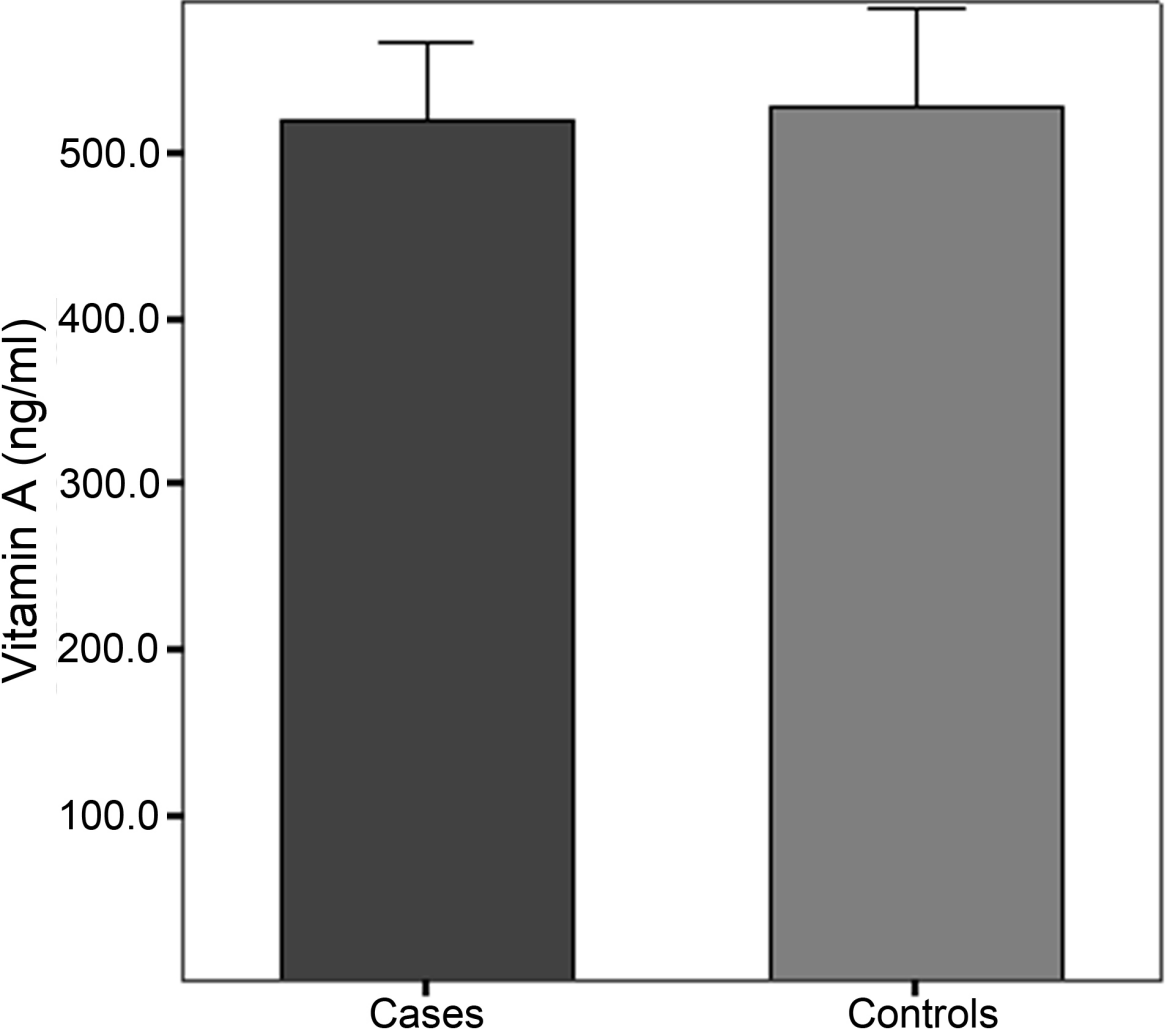
Plasma concentrations of vitamin A (ng/ml) in POAG cases and controls. Differences between means in cases and controls were not statistically significant (p>0.05).

**Figure 2:**
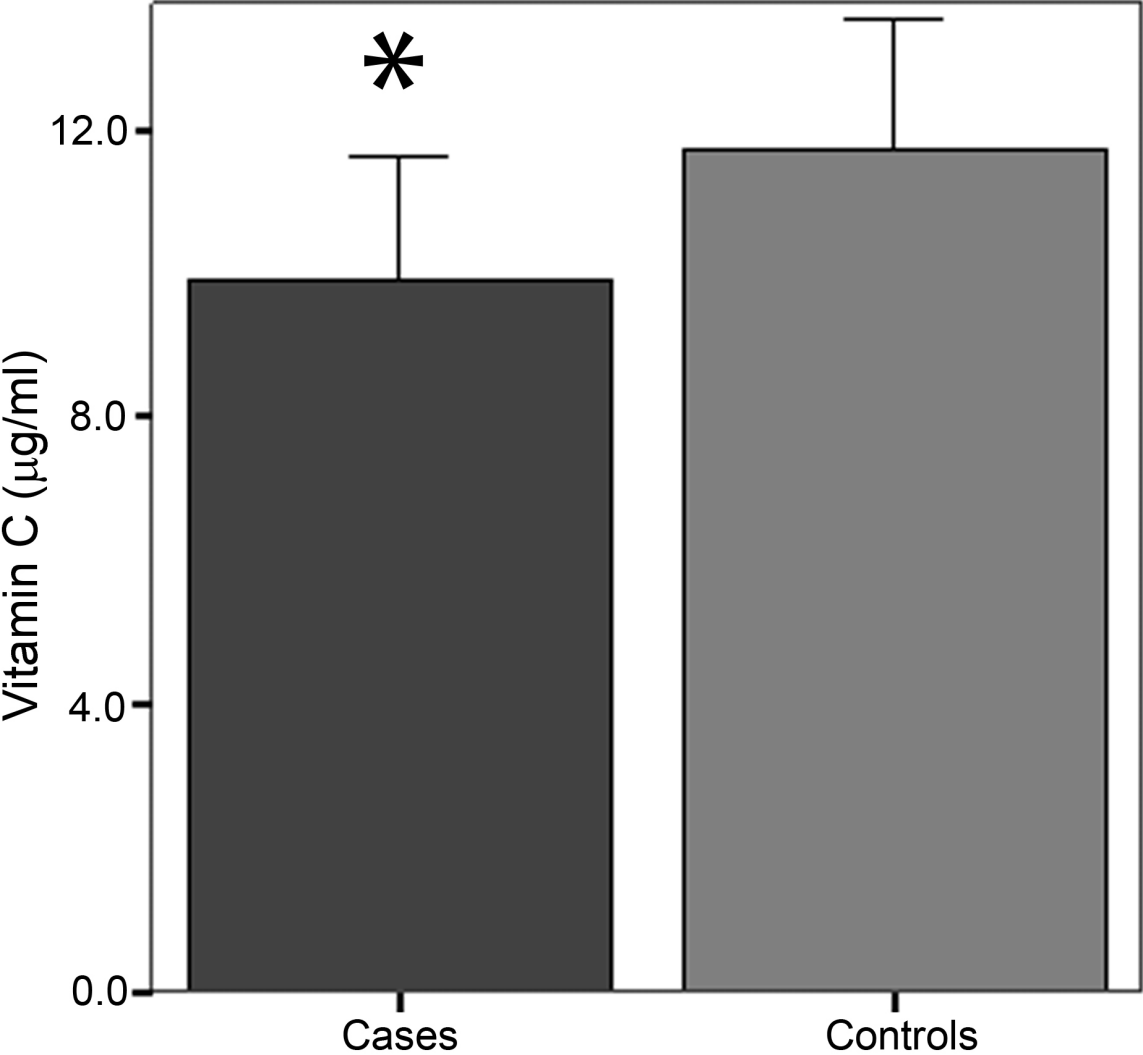
Plasma concentrations of vitamin C (µg/ml) in POAG cases and controls. *Statistically significant (p<0.0125) differences of means between cases and controls.

We also analyzed the plasma concentrations of vitamin C and A for significant differences between cases and controls. Although plasma concentrations of vitamin A tended to be lower in POAG patients than in control subjects, differences did not reach statistical significance (519.3±47.1 ng/ml in POAG cases versus 527.7±58.9 ng/ml in controls; p=0.174; Figure 1). However, plasma concentrations of vitamin C were significantly lower in POAG patients than in controls (9.9±1.7 µg/ml versus 11.7±1.8 µg/ml; p<0.001; Figure 2).

In addition, we tested the polymorphisms in the vitamin C cotransporters for association with plasma vitamin C concentrations (Table 3). In both POAG cases (p<0.001) and controls (p=0.001), we found a strong association between rs1279683 in *SLC23A2* and plasma vitamin C concentrations. We also observed that homozygous subjects for the G allele of rs1279683 in *SLC23A2* (GG) had significantly lower (p<0.001) vitamin C concentrations than carriers of the ancestral allele (AA + AG). This association remained statistically significant (p<0.0125) even after multivariate adjustment for potential confounders (sex, age, BMI, smoking, and drinking). These results agreed with our previous observation when analyzing the association between rs1279683 and POAG risk. Moreover, the association of rs1279683 with higher POAG risk was mediated by the SNP’s association with lower plasma vitamin C concentrations, given that after multivariate adjustment for plasma vitamin C concentrations, rs1279683 did not reach statistical significance in the logistic regression model (OR: 0.73; 95% CI: 0.41–1.29; p=0.277).

**Table 3:** Plasma vitamin A and vitamin C concentrations (mean±SD) depending on the corresponding *RBP1*, *SLC23A1*, or *SLC23A2* genotypes in POAG cases and controls.

** **	** **	** **	** **	**Plasma concentration of vitamins**									
** **	** **	** **	Alleles*	**Cases (n=150)**			** **	** **	**Controls (n=150)**			** **	** **
**Vitamin**	**Gene**	**SNP**	**(1/2)**	**1/1**	**1/2**	**2/2**	**p1**	**p2**	**1/1**	**1/2**	**2/2**	**p1**	**p2**
Vitamin A (ng/ml)	*RBP1*	rs176990	T/G	521.5±44.0	518.9±49.4	518.2±47.1	0.947	0.847	539.8±43.1	528.4±62.6	519.8±60.9	0.361	0.256
Vitamin A (ng/ml)	*RBP1*	rs190910	A/T	516.0±37.3	519.9±50.0	520.9±48.7	0.907	0.804	523.6±51.1	528.2±62.6	528.8±57.3	0.935	0.871
Vitamin C (µg/ml)	*SLC23A1*	rs10063949	T/C	10.0±1.8	10.0±1.7	9.6±1.7	0.527	0.258	11.7±1.7	11.7±1.8	11.8±2.0	0.900	0.673
Vitamin C (µg/ml)	*SLC23A2*	rs1279683	A/G	10.3±1.7	10.6±1.6	9.0±1.4^a,b^	<0.001**	<0.001**	12.0±1.9	12.1±1.8	10.9±1.6^c,d^	0.001**	<0.001**

*Allele 1 is the ancestral allele. Allele 2 is the variant allele. **: Statistically significant p-value (p<0.0125) after the Bonferroni correction for multiple comparisons. p1: Uncorrected p-value obtained in the ANOVA test including the three genotypes. p2: Uncorrected p-value obtained in the Student's  t-test comparing the homozygous subjects for the variant allele (2) with carriers of the ancestral allele (1/1+1/2). Bonferroni post-hoc comparisons: ^a^p-values for comparison of means between vitamin C concentrations in GG subjects versus AA (uncorrected p<0.001;  Bonferroni corrected p<0.001) in POAG cases. ^b^p-values for comparison of means between vitamin C concentrations in GG subjects versus AG (uncorrected p<0.001;  Bonferroni corrected p=0.001) in POAG cases. ^c^p-values for comparison of means between vitamin C concentrations in GG subjects versus AA (uncorrected p=0.010;  Bonferroni corrected p=0.038) in controls. ^d^p-values for comparison of means between vitamin C concentrations in GG subjects versus AG (uncorrected p<0.001;  Bonferroni corrected p=0.001) in controls. No significant differences were found between AA and AT genotypes for vitamin C concentrations.

We did not observe any significant association between the SNP in *SLC23A1* and plasma vitamin C concentrations, or between the SNPs in *RBP1* and plasma vitamin A concentrations (Table 3).

## Discussion

The present study has revealed for the first time a significant association between the rs1279683 (A>G) single-nucleotide polymorphism (SNP) in *SLC23A2* and higher risk of POAG in homozygous carriers for the G allele (GG). The association of POAG risk with this SNP appears to be affected by its influence in determining plasma vitamin C concentrations, as homozygous carriers of the G allele have significantly lower plasma vitamin C concentrations than the other genotypes. As far as we know, this is also the first time that a significant association between the rs1279683 polymorphism in *SLC23A2* and plasma vitamin C concentration has been reported in humans. Previous works have demonstrated the relevance of this gene in animal models and cultured cells [25,26]. Although rs1279683 is an intronic SNP, functional assays of this variant have not been performed and it could be in linkage disequilibrium with the causal variant, if we take into account our observed results in terms of lower plasma vitamin C concentrations in homozygous subjects for the G allele, we can hypothesize that the G allele could be associated with lower expression of *SLC23A2*.

Regarding the rs10063949 polymorphism in *SLC23A1*, we did not find differences in genotype distribution between the POAG patients and the control group. Although both *SLC23A1* and *SLC23A2* codify for two isoforms of the sodium L-ascorbic acid cotransporters, and both have important functions related to ascorbic acid metabolism, *SLC23A1* is expressed in the digestive, reproductive, and urinary system (not in the visual system). In contrast, *SLC23A2* is highly expressed in the eye [27,28], and our results are in agreement with this tissue specificity. On the other hand, genetic variations in the vitamin C transporter *SLC23A2* have been associated with several types of cancer [29-31], supporting the relevance of this gene in antioxidant mechanisms.

Several authors have demonstrated the existence of oxidative stress processes in POAG [32-34]. The cause of this phenomenon is an imbalance between production of free radicals and antioxidant defenses, so that the body is unable to inhibit the cellular damage caused by reactive oxygen species (ROS).

Izzotti et al. [35], by measuring an increase in 8-hydroxy-2'-deoxyguanosine, demonstrated the occurrence of oxidative DNA damage in the trabecular meshwork (TM). In addition, oxidative stress also appears to be related to the neuronal death characteristic of POAG [36]. We observed significantly lower plasma levels of vitamin C in POAG patients than in healthy subjects. These results agree with those of Yuki et al. [20], who found lower serum levels of vitamin C in normal-tension glaucoma patients. Leite et al. [37] studied the ascorbic acid concentration in the secondary aqueous humor of glaucomatous patients, and found that it was approximately twofold lower in comparison with the primary AH of glaucomatous and cataract patients. This reduction in ascorbic acid (vitamin C) concentration in glaucoma patients is logical, since this vitamin has a high antioxidant activity, and subjects with primary open-angle glaucoma lack this capacity.

The other antioxidant vitamin studied, vitamin A, is very important in maintaining ocular health and preventing problems like vision loss, twilight blindness, cataracts, xerophthalmia, and glaucoma [17]. In our study, although the plasma vitamin A concentrations tended to be lower in POAG patients than in controls, this difference was not statistically significant. The higher complexity of vitamin A metabolism over that of vitamin C may contribute to the observed results. In agreement with our results, Yuki et al. [20] did not find statistically significant differences in plasma vitamin A concentration between glaucoma patients and controls. In the present study, we also investigated two SNPs in *RBP1*. RBP1 is one of the carrier proteins involved in the transport of retinol. It is highly expressed in the eye and influences photoreceptor outer segment assembly through a mechanism unrelated to rhodopsin regeneration [38]. However, we did not find significant associations with plasma vitamin A concentration in terms of the studied SNPs, rs176990 and rs190910.

In conclusion, the results of our study support the important role of dietary intake of antioxidants, and certain genetic polymorphisms that modulate their effects, in POAG risk. Specifically, our results show that low plasma concentrations of vitamin C, and SNP rs1279683 in *SLC23A2* through its significant association with low plasma vitamin C concentrations, are POAG risk factors. More nutrigenetic studies are required to confirm this association in other populations, as well as to obtain further knowledge on new gene–diet interactions in POAG pathology.
